# Alternative analyses for handling incomplete follow-up in the intention-to-treat analysis: the randomized controlled trial of balloon kyphoplasty versus non-surgical care for vertebral compression fracture (FREE)

**DOI:** 10.1186/1471-2288-12-35

**Published:** 2012-03-24

**Authors:** Jonas Ranstam, Aleksandra Turkiewicz, Steven Boonen, Jan Van Meirhaeghe, Leonard Bastian, Douglas Wardlaw

**Affiliations:** 1 RC Syd, Skåne University Hospital in Lund, and Department of Clinical Sciences, Lund University, SE-22185 Lund, Sweden; 2 Leuven University Centre for Metabolic Bone Diseases and Division of Geriatric Medicine, Leuven, Belgium; 3 Algemeen Ziekenhuis St-Jan Brugge-Oostende AV, Brugge, Belgium; 4 Klinikum Leverkusen, Leverkusen, Germany; 5 Orthopaedic Department, Woodend Hospital, NHS Grampian, Aberdeen, UK

## Abstract

**Background:**

Clinical trial participants may be temporarily absent or withdraw from trials, leading to missing data. In intention-to-treat (ITT) analyses, several approaches are used for handling the missing information - complete case (CC) analysis, mixed-effects model (MM) analysis, last observation carried forward (LOCF) and multiple imputation (MI). This report discusses the consequences of applying the CC, LOCF and MI for the ITT analysis of published data (analysed using the MM method) from the Fracture Reduction Evaluation (FREE) trial.

**Methods:**

The FREE trial was a randomised, non-blinded study comparing balloon kyphoplasty with non-surgical care for the treatment of patients with acute painful vertebral fractures. Patients were randomised to treatment (1:1 ratio), and stratified for gender, fracture aetiology, use of bisphosphonates and use of systemic steroids at the time of enrolment. Six outcome measures - Short-form 36 physical component summary (SF-36 PCS) scale, EuroQol 5-Dimension Questionnaire (EQ-5D), Roland-Morris Disability (RMD) score, back pain, number of days with restricted activity in last 2 weeks, and number of days in bed in last 2 weeks - were analysed using four methods for dealing with missing data: CC, LOCF, MM and MI analyses.

**Results:**

There were no missing data in baseline covariates values, and only a few missing baseline values in outcome variables. The overall missing-response level increased during follow-up (1 month: 14.5%; 24 months: 28%), corresponding to a mean of 19% missing data during the entire period. Overall patterns of missing response across time were similar for each treatment group. Almost half of all randomised patients were not available for a CC analysis, a maximum of 4% were not included in the LOCF analysis, and all randomised patients were included in the MM and MI analyses. Improved estimates of treatment effect were observed with LOCF, MM and MI compared with CC; only MM provided improved estimates across all six outcomes considered.

**Conclusions:**

The FREE trial results are robust as the alternative methods used for substituting missing data produced similar results. The MM method showed the highest statistical precision suggesting it is the most appropriate method to use for analysing the FREE trial data.

**Trial Registration:**

This trial is registered with ClinicalTrials.gov (number NCT00211211).

## Background

The intention-to-treat (ITT) principle is one of the most important approaches used in performing statistical analyses for randomised clinical trials [1]. It implies that for all patients randomised to a treatment, data should be analysed according to the treatment to which the patient was allocated, irrespective of whether they received this or some other treatment, or no treatment at all. Thus, patients randomised to a control arm who actually receive the treatment under investigation are regarded as 'controls' rather than 'treated cases'; in addition, patients who fail to comply with their assigned treatment are assessed as though the treatment was taken correctly [2]. The purpose of the ITT principle is to preserve the theoretical basis for the validity of the statistical results, specifically by eliminating the possibility that patients with known or unknown prognostic factors are systematically selected to a treatment [1].

Nonetheless, despite being reported as early as the 1960s [3], included in the International Conference on Harmonisation (ICH) Good Clinical Practice (GCP) guidelines [4], and being clearly described in methodological papers specifically targeted at an orthopaedic audience [1], the ITT principle is not well known or uniformly used by orthopaedic investigators. Indeed, a recent systematic review (covering the years 2005-2008) showed that the ITT principle was adhered to in only 96 out of 274 (35%) published orthopaedic randomised trials [5].

It is not uncommon for patients participating in clinical trials to be temporarily absent from follow-up visits or terminate their involvement. When this happens, some or all information on the treatment outcome for these patients will not be available for the statistical analysis. Depending on the statistical method used, several approaches are available for handling the missing information in the ITT analysis. Either the available information can be analysed in its current form, or the missing data can be replaced with hypothetical observations (imputed data) to allow patients for whom some data are missing to be included in the analysis. The method used to adjust for missing data should be chosen appropriately, depending on the reason for the missing data.

By imputing data (i.e. filling in missing values with reasonable data), patients with some missing information can be included in an analysis. In fact, inclusion of patients with complete information only may not represent a randomly selected subset of the overall randomized patient population. A complete-case (CC) analysis includes only those patients with both baseline and corresponding follow-up outcome values and, as such, is at variance with the ITT principle (which requires that all cases, whether complete or incomplete, are included in the analysis) [2]. An alternative for including all patients is to perform a mixed-effects model (MM) analysis. By modelling both random and fixed effects, this technique can be used to analyse patients with incomplete follow-up.

Missing data can be imputed via simple or multiple imputation methodology. One of the most frequently used simple imputation techniques is last observation carried forward (LOCF). In this method, the last observed values for a patient are used in place of the missing values; this method assumes that the last known state in the study represents the patient's true outcome [2]. This implicit assumption of representativity (which is present in all simple imputation techniques) may be considered a weakness, and should be questioned when the treatment effect changes during follow-up. Furthermore, simple imputation techniques are deterministic and lead to an underestimation of the variability in the outcome.

In contrast, multiple imputation (MI) is a stochastic technique which depends on model-based imputation of multiple values for each missing observation. The values are combined using the technique described by Rubin in 1987 [6]. Thus, MI provides a superior alternative to simple imputation. It does not underestimate variability, and the models for imputation and efficacy analysis can be developed independently, thereby increasing the realism of the assumptions underlying the imputation [2].

The advantages provided by the most recently developed techniques, such as MM and MI (compared with CC and LOCF), are also associated with much greater complexity. As a consequence, widespread application of these techniques may be delayed because those unfamiliar with the methodology could have concerns about the validity of the results generated. Thus, further discussion about the importance of the ITT principle, along with greater understanding of the problems associated with missing values in orthopaedic randomised trials, is required.

The aim of this report is to describe and discuss the consequences of applying the three alternative methods (CC, LOCF and MI) for the ITT analysis of the Fracture Reduction Evaluation (FREE) trial - a randomized controlled trial of balloon kyphoplasty (BKP) versus non-surgical care for vertebral compression fracture, analysed using the MM method and recently published in The Lancet [7].

## Materials and methods

### The Fracture Reduction Evaluation (FREE) trial

Detailed methodology for the FREE trial has been presented previously [7]. In brief, the FREE trial was a randomised, non-blinded trial comparing non-surgical care alone with BKP for the treatment of patients with acute painful vertebral fractures. The study included patients from 21 sites in eight countries (Austria, Belgium, France, Germany, Italy, Sweden, the Netherlands and the United Kingdom) and was conducted from February 2003 through December 2005.

All participants had at least one acute thoracic or lumbar (T5-L5) vertebral fracture with bone marrow signal changes on magnetic resonance imaging (MRI), and vertebral height reduction (> 15% of predicted vertebral height) compared with the adjacent vertebrae. Painful vertebral fractures were diagnosed by the local investigator; up to three fractures could be treated if they also had signal changes, rapidly progressive height loss or pseudoarthrosis.

Participants had self-assessed back pain of at least 4 on a scale from 0 (no pain) to 10 (worst pain imaginable) that started within the past 3 months and was not attributable to other causes. Vertebral fractures were included irrespective of aetiology; however, fractures due to primary bone tumours, osteoblastic metastases or high-energy trauma were excluded. Participants gave written informed consent before enrolment, and the protocol and consent forms were approved by local ethics committees. The trial was conducted in accordance with the Declaration of Helsinki, and is registered with ClinicalTrials.gov (number NCT00211211).

Patients were randomly assigned in a 1:1 ratio to receive BKP or non-surgical care using a computer-generated schedule. Study randomisation was stratified for gender, fracture aetiology, use of bisphosphonates at the time of enrolment and use of systemic steroids during the last 12 months before enrolment, but not for number of prevalent fractures per participant. A permuted block randomisation (stratified as indicated) was generated using PROC PLAN prior to the start of the study.

Percutaneous BKP was performed with introducer tools, inflatable bone tamps, and polymethylmethacrylate bone cement and delivery devices (Medtronic Spine LLC, Sunnyvale, CA, USA) using a bilateral, transpedicular or extrapedicular approach. Patients received analgesics, bed rest, bracing, physiotherapy, rehabilitation programmes and walking aids according to the standard practices of participating physicians and hospitals. All patients were referred for treatment with calcium and vitamin D supplements, and antiresorptive or anabolic agents.

The primary endpoint was the change from baseline to 1 month in quality of life (QoL) assessed using the Short-form 36 (SF-36) physical component summary (PCS) scale. Secondary endpoints included: EuroQol 5-Dimension Questionnaire (EQ-5D); SF-36 subscale scores; function measured using the Roland-Morris Disability (RMD) score; back pain assessed with a visual-analogue scale (VAS; scale 0-10); limited days of activity and bed rest because of back pain during the previous 2 weeks; and patient satisfaction assessed on a 20-point Likert scale (extremely dissatisfied to extremely satisfied). Outcomes were assessed at baseline/screening and at 1, 3, 6, 12 and 24 months; back pain was also assessed at 7 days.

### Statistical methods

Six outcome measures - SF-36 PCS scale, EQ-5D, RMD score, back pain, number of days with restricted activity in last 2 weeks and number of days in bed in last 2 weeks - were analysed using four methods for dealing with missing data: CC analysis, simple imputation with LOCF analysis, MM analysis on all available data and MI analysis.

The CC analysis included only patients with both baseline and all follow-up values for respective outcomes. For the LOCF analysis, only patients with available baseline values were included; missing follow-up values were replaced by the patient's last observed value, based on the assumption that this represented the treatment effect. In contrast with the LOCF method, MI is a stochastic imputation method based on the assumption that missing values can be replaced with values generated by a model incorporating random variation. The generation of such values is performed repeatedly providing a series of complete datasets. These datasets are then analysed using standard methods for complete data, and the results are combined to provide a set of parameter estimates and their standard errors, from which confidence intervals and p-values can be derived. The MI model can be different from the model used for the final data analysis. In this study we imputed data using as-treated models, and analysed them according to ITT [2].

For the CC, LOCF and MI methods, the analysis was performed using a conventional repeated-measures ANOVA design. The model included treatment group and visit as fixed factors, as well as their interaction, together with covariates representing the randomisation stratification factors (gender, fracture aetiology, use of bisphosphonates at the time of enrolment and use of systemic steroids during the last 12 months before enrolment) and baseline values.

In the MM analysis, all patients with at least one baseline or follow-up value were included. An MM analysis includes both fixed and random factors: in the current analysis, treatment group and visit were included as fixed factors, and patient was included as a random factor. The model included interactions between treatment and visit. Randomisation stratification factors (gender, fracture aetiology, use of bisphosphonates at the time of enrolment and use of systemic steroids during the last 12 months before the time of enrolment) and baseline value were included as covariates. Compound symmetry structure for covariance between measurements was assumed. Maximum restricted likelihood procedure was used to fit the model and denominator degrees of freedom were estimated using Satterthwaite's approximation. This mixed model analysis is, with balanced data, equivalent to the conventional repeated measures ANOVA with sphericity assumption [8].

#### Assumptions

The CC approach assumes that data are missing completely at random (MCAR) - i.e. that missing data are a random subsample of all data. The LOCF approach assumes that baseline values are MCAR, and that there is no change in treatment effect during follow-up. It is also assumed that there is no variation in the measurement of treatment effect itself. In both the MM analysis and the MI technique, it is assumed that data are missing at random (MAR), meaning that the probability that an observation is missing may depend on observed data but not on missing data. Thus, the likelihood function for the complete dataset, with respect to inference on the unknown parameters that characterise the complete data distribution, is the same as the likelihood function for the observed data. In the MM approach, the MAR assumption needs to be fulfilled in the analysis model, whereas in the MI approach only the imputation model needs to satisfy the MAR assumption.

#### Imputation model

In this study, the imputation model included all six outcomes at baseline and follow-up visits, all randomisation stratification factors, age, treatment centre and number of fractures at baseline (≥ 1), in addition to treatment 'as received'. Treatment centres with less than 7% of patients overall were combined. Treatment 'as received', rather than treatment 'as randomised', was used in the imputation model to recover the 'true' outcome values for patients. This was done because the observed outcome values for the patient are influenced by the treatment received not the treatment assigned.

Multiple imputation using fully conditional specification (or 'chained equations') [9] was used to create 30 imputed datasets, primarily because the distribution of number of days with reduced activity and number of days in bed were substantially non-normal. Therefore, the variables were treated as ordinal in the imputation model. Other outcome variables were treated as continuous. PCS followed a normal distribution and was not transformed. Roland-Morris disability score, VAS and EQ-5D were transformed to normality using Box-Cox transformation and imputed with predictive mean matching method [10] to ensure that the imputed values did not exceed the natural range of values for those variables. After imputation the values were transformed back to the original scale. Other covariates were included as dummy (0-1) variables. To create the imputation in STATA the imputation by chained equations (ICE) implementation was used [11].

The MI approach provides the means to estimate the fraction of missing information relative to the complete information for each parameter of interest.([6], section 3.3) If the missing data do not provide any additional information about the parameter of interest to that which can be observed from the available data then the fraction will be equal to zero. If the missing data contain a high proportion of information, and this is not also contained within the observed data, then the fraction of missing information will be high.

## Results

### Missing response

**Figure 1:**
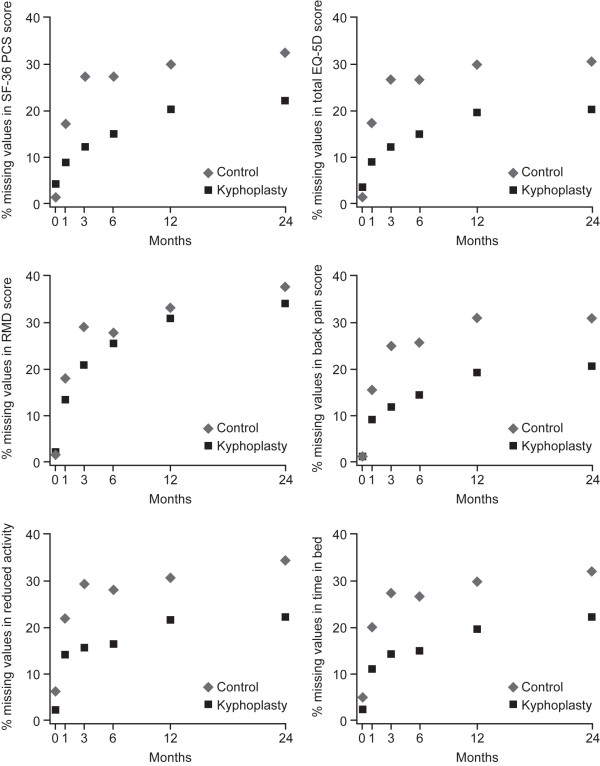
**Missing data by treatment group in the Fracture Reduction Evaluation (FREE) trial**. EQ-5D, EuroQol 5-dimension; RMD, Roland-Morris Disability; SF-36 PCS, short form-36 physical component summary.

There were no missing data in baseline covariates values, and only a few missing baseline values in outcome variables. The missing-response level increased during follow-up from an average of 14.5% at 1 month to 28% at 24 months; corresponding to a mean of 19% missing data during the entire follow-up period. Of the six outcome variables, back pain had the highest available response level (83.4%) and RMD score had the lowest (77.2%; Table 1). Overall patterns of missing response across time were similar in both treatment groups, with consistently higher available response in the BKP group than in the control group (mean 9% higher for all outcome variables, with the exception of the RMD score where the difference between treatments was approximately 3%; Figure 1).

**Table 1:** Percentage missing data in the FREE trial overall

	Follow-up (time after randomisation)						
**Measure**	**Baseline**	**1 month**	**3 months**	**6 months**	**12 months**	**24 months**	**Mean**

SF-36 PCS	2.7	13.0	19.7	21.0	25.0	27.3	18.1

EQ-5D	2.3	13.0	19.3	20.7	24.7	25.3	17.6

RMD score	1.7	15.7	25.0	26.7	32.0	36.0	22.8

Back pain	0.7	12.0	18.0	19.7	24.7	24.3	16.6

Restricted activity	4.0	18.0	22.3	22.0	26.0	28.3	20.1

Days in bed	3.3	15.3	20.7	20.7	24.7	27.0	18.6

Values shown are percentages

EQ-5D, EuroQol 5-dimension; RMD, Roland-Morris Disability; SF-36 PCS, short form-36 physical component summary

### Estimated fraction of missing information

The fraction of missing information can be used instead of the traditional percentage of missing values to assess the level of missing response in surveys [12]. It does not need to be equal to the nominal amount of missing values, but can be smaller if there is information about missing data in other correlated and observed variables, or larger if patients with missing values have more influence on regression coefficients than patients with observed values. The fraction of missing information and nominal percentage of missing values for each of the six outcomes are presented in Table 2. With the exception of 'days in bed', the fraction of missing information estimated using the MI method was lower than the nominal percentage of missing data per visit.

**Table 2:** Fraction of missing information compared with the nominal amount of missing information

	Follow-up period									
	**1 month**		**3 months**		**6 months**		**12 months**		**24 months**	

**Outcome**	**FMI**	**MD**	**FMI**	**MD**	**FMI**	**MD**	**FMI**	**MD**	**FMI**	**MD**

SF-36 PCS	11.9	15.7	15.8	22.3	17.2	23.7	23.9	27.7	30.1	30.0

EQ-5D	14.5	15.3	13.1	21.7	18.0	23.0	18.4	27.0	20.9	27.7

RMD	9.9	17.3	19.8	26.7	18.2	28.3	21.0	33.7	19.9	37.7

Back pain	12.3	12.7	15.7	18.7	19.0	20.3	17.3	25.3	29.6	25.0

Restricted activity	9.6	22.0	14.0	26.3	19.6	26.0	19.3	30.0	20.0	32.3

Days in bed	21.2	18.7	24.6	24.0	27.5	24.0	35.4	28.0	34.3	30.3

Values shown are percentages

EQ-5D, EuroQol 5-dimension; FMI, fraction of missing information; RMD, Roland-Morris Disability; SF-36 PCS, short form-36 physical component summary; MD, nominal amount of missing data in baseline and follow-up data

### Comparison of analysis methods

Results for the analysis of variance with repeated measures on the CC dataset, the dataset imputed with LOCF method, and datasets resulting from the MI procedure and MM analysis on all available data are shown in Table 3. Complete-case analyses were performed on 62%, 64%, 49%, 62%, 54% and 66% of patients for SF-36 PCS, EQ-5D, RMD score, number of days in bed, number of days with restricted activity, and back pain, respectively. Almost half of all randomised patients were not available for a CC analysis. A maximum of 4% of patients were not included in the LOCF analysis because of missing baseline values. All randomised patients were included in the MM and MI analyses.

**Table 3:** Comparison of methods for handling missing data for ITT analysis of the FREE trial

	Complete case analysis				Last observation carried forward				Mixed effects model				Multiple imputation			
**Measure**	**EE**	**SE**	**95% CI**	**P value**	**EE**	**SE**	**95% CI**	**P value**	**EE**	**SE**	**95% CI**	**P value**	**EE**	**SE**	**95% CI**	**P value**

**SF-36 PCS**																

1 month follow-up	**8.00**	1.3724	[5.31,10.69]	0.0000	**5.24**	1.0320	[3.22,7.27]	0.0000	**5.32**	1.1139	[3.14,7.51]	0.0000	**5.09**	1.1509	[2.83,7.34]	0.0000

3 months follow-up	**5.60**	1.3724	[2.91,8.29]	0.0000	**4.52**	1.0320	[2.50,6.54]	0.0000	**4.13**	1.1453	[1.89,6.38]	0.0003	**4.06**	1.1778	[1.75,6.37]	0.0006

6 months follow-up	**4.16**	1.3724	[1.47,6.85]	0.0025	**4.06**	1.0320	[2.04,6.08]	0.0001	**3.39**	1.1482	[1.13,5.64]	0.0032	**3.39**	1.1853	[1.06,5.71]	0.0043

12 months follow-up	**2.07**	1.3724	[-0.62,4.76]	0.1315	**2.92**	1.0320	[0.90,4.95]	0.0046	**1.70**	1.1647	[-0.59,3.98]	0.1451	**2.42**	1.2333	[0.00,4.84]	0.0499

24 months follow-up	**2.49**	1.3724	[-0.20,5.18]	0.0694	**2.81**	1.0320	[0.78,4.83]	0.0065	**1.68**	1.1757	[-0.63,3.98]	0.1538	**2.40**	1.2855	[-0.12,4.92]	0.0617

Overall treatment effect	**4.47**	1.1027	[2.30,6.63]	0.0001	**3.91**	0.8497	[2.24,5.58]	0.0000	**3.24**	0.8979	[1.48,5.00]	0.0003	**3.47**	0.9264	[1.66,5.29]	0.0002

**EQ5D**																

1 month follow-up	**0.20**	0.0429	[0.12,0.28]	0.0000	**0.18**	0.0378	[0.10,0.25]	0.0000	**0.17**	0.0377	[0.10,0.24]	0.0000	**0.16**	0.0397	[0.09,0.24]	0.0000

3 months follow-up	**0.10**	0.0429	[0.02,0.18]	0.0195	**0.15**	0.0378	[0.07,0.22]	0.0001	**0.11**	0.0387	[0.03,0.18]	0.0054	**0.10**	0.0394	[0.03,0.18]	0.0090

6 months follow-up	**0.11**	0.0429	[0.02,0.19]	0.0130	**0.17**	0.0378	[0.09,0.24]	0.0000	**0.12**	0.0389	[0.04,0.20]	0.0019	**0.11**	0.0405	[0.03,0.19]	0.0065

12 months follow-up	**0.11**	0.0429	[0.02,0.19]	0.0125	**0.15**	0.0378	[0.08,0.23]	0.0000	**0.10**	0.0394	[0.02,0.18]	0.0097	**0.10**	0.0406	[0.02,0.18]	0.0131

24 months follow-up	**0.09**	0.0429	[0.00,0.17]	0.0451	**0.13**	0.0378	[0.05,0.20]	0.0007	**0.08**	0.0395	[0.00,0.16]	0.0497	**0.07**	0.0411	[-0.01,0.15]	0.0909

Overall treatment effect	**0.12**	0.0339	[0.05,0.19]	0.0004	**0.15**	0.0319	[0.09,0.22]	0.0000	**0.12**	0.0302	[0.06,0.18]	0.0001	**0.11**	0.0302	[0.05,0.17]	0.0003

**RMD score**																

1 month follow-up	-**4.45**	0.8743	[-6.16, -2.74]	0.0000	**-3.52**	0.6573	[-4.81,-2.23]	0.0000	**-4.22**	0.6793	[-5.55,-2.89]	0.0000	**-3.86**	0.6845	[-5.20, -2.52]	0.0000

3 months follow-up	-**2.86**	0.8743	[-4.58,-1.15]	0.0011	**-3.48**	0.6573	[-4.77,-2.19]	0.0000	**-3.64**	0.7034	[-5.01,-2.26]	0.0000	**-3.30**	0.7319	[-4.74, -1.87]	0.0000

6 months follow-up	-**2.04**	0.8743	[-3.75,-0.32]	0.0198	**-3.07**	0.6573	[-4.36,-1.78]	0.0000	**-2.96**	0.7072	[-4.35,-1.58]	0.0000	**-2.76**	0.7258	[-4.19, -1.34]	0.0001

12 months follow-up	-**1.95**	0.8743	[-3.66,-0.24]	0.0258	**-2.99**	0.6573	[-4.28,-1.70]	0.0000	**-2.81**	0.7207	[-4.22,-1.40]	0.0001	**-2.50**	0.7409	[-3.95, -1.05]	0.0007

24 months follow-up	-**0.60**	0.8743	[-2.31,1.12]	0.4945	**-2.34**	0.6573	[-3.62,-1.05]	0.0004	**-1.43**	0.7327	[-2.87,0.00]	0.0506	**-1.65**	0.7431	[-3.10, -0.19]	0.0267

Overall treatment effect	-**2.38**	0.7355	[-3.82,-0.94]	0.0012	**-3.08**	0.5667	[-4.19,-1.97]	0.0000	**-3.01**	0.5716	[-4.13,-1.89]	0.0000	**-2.81**	0.5737	[-3.94, -1.69]	0.0000

**VAS score**																

1 month follow-up	**-1.92**	0.3329	[-2.57,-1.27]	0.0000	**-1.87**	0.2807	[-2.42,-1.32]	0.0000	**-1.89**	0.2872	[-2.46,-1.33]	0.0000	**-1.76**	0.3064	[-2.36,-1.16]	0.0000

3 months follow-up	**-1.51**	0.3329	[-2.16,-0.86]	0.0000	**-1.71**	0.2807	[-2.26,-1.16]	0.0000	**-1.53**	0.2958	[-2.11,-0.95]	0.0000	**-1.35**	0.3120	[-1.96,-0.74]	0.0000

6 months follow-up	**-1.53**	0.3329	[-2.18,-0.88]	0.0000	**-1.76**	0.2807	[-2.31,-1.21]	0.0000	**-1.56**	0.2980	[-2.15,-0.98]	0.0000	**-1.43**	0.3181	[-2.05,-0.80]	0.0000

12 months follow-up	**-0.77**	0.3329	[-1.42,-0.11]	0.0214	**-1.31**	0.2807	[-1.86,-0.76]	0.0000	**-0.93**	0.3055	[-1.53,-0.33]	0.0023	**-0.85**	0.3153	[-1.47,-0.23]	0.0071

24 months follow-up	**-0.77**	0.3329	[-1.42,-0.12]	0.0203	**-1.14**	0.2807	[-1.69,-0.59]	0.0000	**-0.80**	0.3043	[-1.39,-0.20]	0.0089	**-0.61**	0.3408	[-1.28,0.06]	0.0725

Overall treatment effect	**-1.42**	0.2316	[-1.87,-0.96]	0.0000	**-1.65**	0.2123	[-2.07,-1.23]	0.0000	**-1.49**	0.2000	[-1.88,-1.10]	0.0000	**-1.20**	0.2248	[-1.64,-0.76]	0.0000

**Resticted activity**																

1 month follow-up	**-4.44**	0.9193	[-6.24,-2.63]	0.0000	**-2.95**	0.6979	[-4.31,-1.58]	0.0000	**-3.46**	0.7466	[-4.93,-2.00]	0.0000	**-3.13**	0.7356	[-4.57,-1.69]	0.0000

3 months follow-up	**-3.79**	0.9193	[-5.59,-1.98]	0.0000	**-3.82**	0.6979	[-5.19,-2.45]	0.0000	**-3.94**	0.7649	[-5.44,-2.44]	0.0000	**-3.59**	0.7542	[-5.07,-2.11]	0.0000

6 months follow-up	**-3.00**	0.9193	[-4.80,-1.19]	0.0011	**-2.96**	0.6979	[-4.32,-1.59]	0.0000	**-2.43**	0.7647	[-3.93,-0.93]	0.0015	**-2.64**	0.7804	[-4.17,-1.11]	0.0007

12 months follow-up	**-1.48**	0.9193	[-3.28,0.32]	0.1067	**-2.64**	0.6979	[-4.00,-1.27]	0.0002	**-2.04**	0.7788	[-3.56,-0.51]	0.0089	**-1.86**	0.7766	[-3.38,-0.34]	0.0167

24 months follow-up	**-1.56**	0.9193	[-3.36,0.24]	0.0896	**-2.17**	0.6979	[-3.54,-0.80]	0.0019	**-1.24**	0.7905	[-2.79,0.31]	0.1156	**-1.62**	0.7800	[-3.15,-0.09]	0.0378

Overall treatment effect	**-2.85**	0.6589	[-4.14,-1.56]	0.0000	**-2.90**	0.5328	[-3.95,-1.86]	0.0000	**-2.62**	0.5357	[-3.67,-1.57]	0.0000	**-2.57**	0.5189	[-3.59,-1.55]	0.0000

**Days in bed**																

1 month follow-up	**-2.71**	0.4710	[-3.63,-1.78]	0.0000	**-2.53**	0.4897	[-3.49,-1.57]	0.0000	**-2.55**	0.4418	[-3.42,-1.69]	0.0000	**-2.45**	0.5682	[-3.56,-1.34]	0.0000

3 months follow-up	**-0.86**	0.4710	[-1.78,0.07]	0.0693	**-1.39**	0.4897	[-2.35,-0.43]	0.0044	**-0.96**	0.4557	[-1.86,-0.07]	0.0342	**-1.48**	0.5809	[-2.62,-0.34]	0.0109

6 months follow-up	**-0.31**	0.4710	[-1.23,0.62]	0.5155	**-0.87**	0.4897	[-1.83,0.09]	0.0757	**-0.30**	0.4565	[-1.19,0.60]	0.5143	**-0.83**	0.5929	[-1.99,0.33]	0.1606

12 months follow-up	**-0.08**	0.4710	[-1.00,0.85]	0.8701	**-0.67**	0.4897	[-1.63,0.29]	0.1741	**0.17**	0.4672	[-0.74,1.09]	0.7113	**-0.35**	0.6261	[-1.57,0.88]	0.5800

24 months follow-up	**-0.64**	0.4710	[-1.57,0.28]	0.1718	**-0.83**	0.4897	[-1.79,0.13]	0.0899	**-0.70**	0.4739	[-1.63,0.23]	0.1393	**-1.21**	0.6211	[-2.42,0.01]	0.0521

Overall treatment effect	**-0.92**	0.3002	[-1.51,-0.33]	0.0022	**-1.26**	0.3894	[-2.02,-0.49]	0.0012	**-0.87**	0.2908	[-1.44,-0.30]	0.0028	**-1.26**	0.3629	[-1.97,-0.55]	0.0005

EE, estimated effect; EQ5D, EuroQol 5-dimension; RMD, Roland-Morris Disability; SE, standard error; SF-36, short form 36 health survey; VAS, visual analogue scale

**Figure 2:**
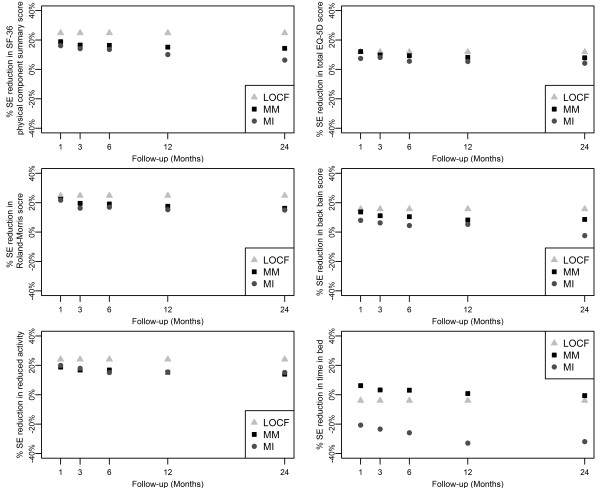
**Percentage reduction in standard error for all follow-up visits for six outcomes**. Five outcomes assessed were: short form-36 physical component summary, EuroQol 5-dimension, Roland-Morris Disability, visual analogue scale and restricted activity. CC, complete case; LOCF, last observation carried forward; MI, multiple imputation; MM, mixed-effects model; SE, standard error.

**Figure 3:**
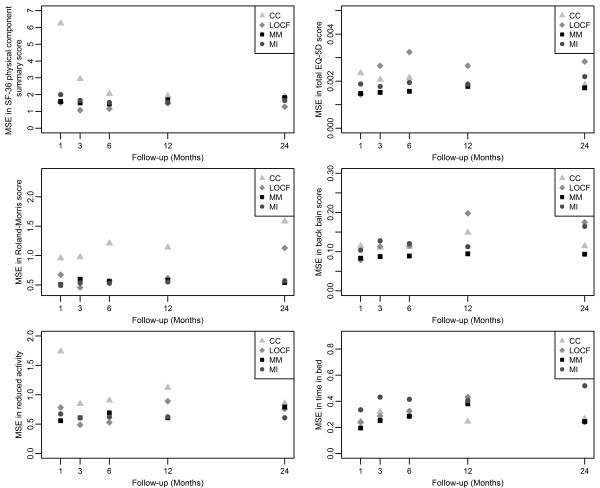
**Mean squared error for all follow-up visits for six outcomes, mean of the estimates from all 4 methods used as reference value**. Five outcomes assessed were: short form-36 physical component summary, EuroQol 5-dimension, Roland-Morris Disability, visual analogue scale and restricted activity. CC, complete case; LOCF, last observation carried forward; MI, multiple imputation; MM, mixed-effects model; SE, standard error.

The consequences for these approaches are, *inter alia*, differences in size of standard errors of the estimates. The standard errors (SE) were compared with those from CC, which was used as the reference method. The mean squared errors (MSE) from CC, LOCF, MM and MI were calculated with means of the estimates from all four methods as reference values. The percentage reductions in size of SE and MSE in estimates of treatment effect at five follow-up periods are presented in Figure 2 and 3.

The highest percentage reductions in standard errors at all follow-up months were observed with the LOCF analysis, about 20% for all outcomes apart from days in bed; MM showed higher (3% on average) percentage reductions in standard errors than MI at each follow-up month. However, it should be noted that LOCF has the potential of underestimating the true standard error, as it assumes no variation in the observations carried forward, so it may not be as reliable as the other two imputation methods.

**Figure 4:**
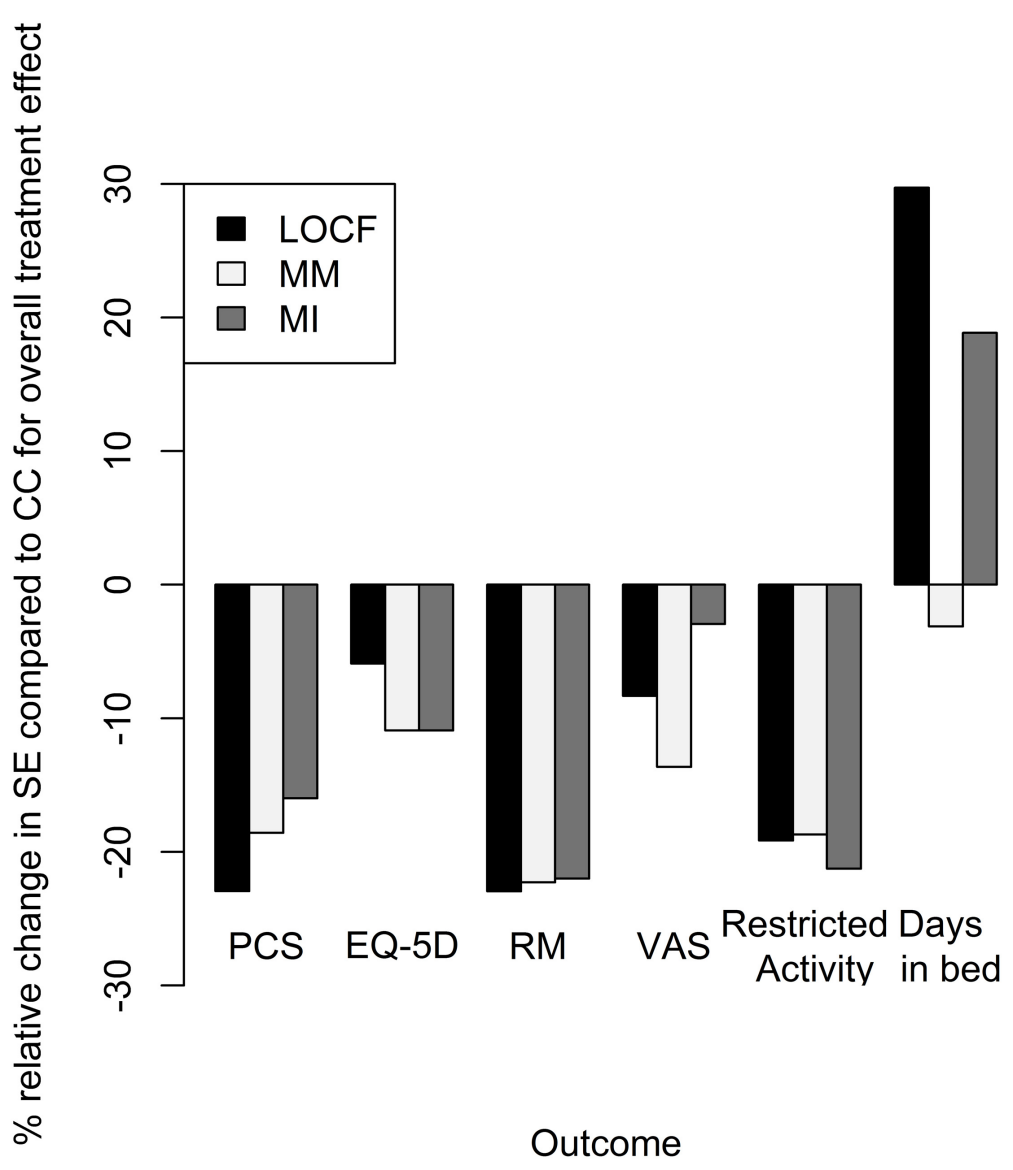
**Percentage relative change in standard error for overall treatment effect in six outcomes**. Six outcomes assessed were: short form-36 physical component summary, EuroQol 5-dimension, Roland-Morris Disability, visual analogue scale, restricted activity and days in bed. CC, complete case; LOCF, last observation carried forward; MI, multiple imputation; MM, mixed-effects model; SE, standard error.

Percentage reductions in size of standard errors compared with CC for overall treatment effect are presented in Figure 4. The reductions achieved when using the imputation methods (LOCF, MM, and MI) allow more precise estimates of the treatment effect to be generated for each outcome. For five of the outcomes, all three methods provided percentage reductions in standard error compared with CC for overall treatment effect (Figure 4). However, for the outcome 'days in bed' only the MM method provided a reduction in size of standard errors, between 1% and 6% (Figures 2 and 4). A reduction was observed at most timepoints (months 1-12) with the MM method, whereas increases in standard error were observed across all follow-up timepoints (months 1-24) for LOCF and MI methods (Figure 2).

These findings are supported by the comparisons of MSEs (Figure 3), which also show that the MM and MI approaches tend to yield more precise estimates, whereas the CC analysis tend to result in lower precision, at least for other outcomes than "days in bed".

## Discussion

The different analysis methods (CC, LOCF, MM and MI) applied to the FREE trial data produced similar results, with only minor differences in standard error sizes observed between the groups. This suggests that the FREE trial data are not dependent on the ITT analysis method used. Indeed, the information contained within the missing data would seem to be of a similar nature to the actual documented information. This implies that the conclusions made about the efficacy of BKP treatment in the FREE trial are robust.

The literature shows that use of the ITT principle in orthopaedic randomized clinical trials (RCT) is limited; in a survey of eight leading orthopaedic journals only 35% of RCT used the ITT method [5]. Thus, lack of ITT use could be a potential source of bias for a comparatively large proportion of orthopaedic RCT. Similar investigations have previously been performed for the general medical literature: an analysis of four leading medical journals demonstrated that 119 out of 249 (48%) RCT published in 1997 used the ITT principle [13], while a later analysis of ten medical journals showed that 249 out of 403 (62%) RCT published in 2002 used the ITT method [14]. Therefore, while the ITT principle appears to be more commonly used in general medical RCT compared with orthopaedic RCT, there is still scope for improvement.

In the FREE study, the missing response level was 19% for the entire 24-month follow-up period. This proportion was similar to that observed for other long-term studies; in a survey of RCT published in eight orthopaedic journals the mean rate of patients lost to follow up was 17% for thirty studies with a follow-up period longer than 1 year [5]. Indeed, increased missing response levels in RCT may be expected over longer time periods; in the same survey of orthopaedic RCT a significant increase in the proportion of patients with missing data was observed over longer follow-up periods [5].

While each imputation method provided similar results, there are certain characteristics related to each analysis method that need to be acknowledged. For example, the CC analysis (which is at variance with the ITT principle) is generally considered insufficient for evaluating data from clinical trials. Data can be missing in a sporadic manner across several different covariates and this can lead to the omission of a high proportion of patients for a CC analysis. Indeed, in the FREE trial almost half of all randomised patients were not available for the CC analysis. It is possible that only including patients with complete information may not provide a representative randomly selected subset of the total randomised population. The higher MSE for the CC analysis can also be interpreted as a sign of bias in the treatment effect estimate. However, in the FREE study the results obtained for the CC analysis were reasonably consistent with the MM, not being an imputation method, and MI suggesting those patients with complete information were representative of the randomised population.

The Cochrane Musculoskeletal Group recommends that imputations based on methods such as LOCF are acceptable in both 'platinum' and 'gold' level publications included in Cochrane systematic reviews [15]. Implicit in the LOCF method is the assumption that the outcome remains constant from the last observed value after drop out and that no measurement errors exist, otherwise there is a risk for bias particularly over longer follow-up periods. In the FREE trial treatment effects were actually observed to change during the follow-up period [7]. At 1 month follow-up, patients treated with BKP showed significantly (p < 0.0001) greater improvements in SF-36 PCS score compared with those who received non-surgical treatment. However, at 12-months follow-up the difference between the two groups had diminished likely due to fracture healing in patients who had received non-surgical treatment. Therefore, use of LOCF to analyze the FREE trial data could be a potential source of bias. However, the present analysis showed that the LOCF method produced similar results to the MM and MI methods suggesting that any potential effects on bias would likely be minimal.

The MI method does not have the disadvantages associated with single imputation methods: variance is not underestimated and treatment effect changes are preserved over time. The MI method does rely to a great extent on the imputation model being correct, but providing the data are normally distributed or can be transformed to normality there is a sound theoretical background to how the imputations are generated [16]. However, if some of the imputed variables cannot be treated as normally distributed then other MI methods, such as chained equations, need to be used. The chained equations method has produced accurate imputations in various settings [9], but the overall theory that proves its correctness is currently being developed. The inclusion of more variables in the imputation model than in the analysis model increases the probability that MAR assumptions hold, although this may lead to an increase in the standard errors of the final estimates [16]. In case the imputation model and the analysis models differ it is crucial to ensure that the models are congenial, i.e. that all variables used in the analysis model are included in the imputation model [17].

When surgical intervention in one group is compared with a non-surgical treatment in another, the proportions of patients lost to follow up could be expected to differ between the groups. The phenomenon has been described earlier [5], and it occurs also in this trial. The higher rate of missing data in the control group may undermine a MAR assumption, which would affect both the MM and CC analysis. However, if the MAR assumption holds for the analysis model used then MI should converge to MM as the number of imputations goes to infinity [18]. For the FREE data there were no substantial differences observed between the MM and MI analyses despite the fact that the imputation model included more variables than the analysis model. This indicates that the MAR assumption holds for the analysis model and that the MM analysis method appears to be optimal.

In the present analysis of the FREE data, the MM method showed the highest statistical precision without the unsubstantiated assumptions required for LOCF. This was particularly evident when the three methods (LOCF, MM and MI) were used to examine the percentage reduction in standard error compared with CC for overall treatment effect. Only MM provided a reduction in variance across all six outcomes measured (including 'days in bed'). The MI method also provided low variance but was not as precise as the MM method across all outcomes considered. This suggests that application of the MM method is probably the most accurate approach for analysing these data. This suggestion is also supported by a simulation study [18].

## Conclusions

The FREE trial results are robust as the four alternative methods used for substituting missing data produced similar results. The small differences observed for the MM and MI models provide strong support for their use. By comparison the CC model included only approximately half of the ITT population and LOCF appeared to overestimate precision, which could potentially produce biased results.

## Competing interests

JR and AT are employed by RC Syd (formerly NKO, Swedish National Musculoskeletal Competence Centre) an organisation that has received compensation for their work from Medtronic Inc. SB has received honoraria for consulting from Kyphon and Medtronic Spine LLC, and has received research funding or grant support from Amgen, Eli Lilly, Kyphon, Medtronic Spine LLC, Merck, Novartis, Procter and Gamble, Sanofi-Aventis, Servier, and Roche-GlaxoSmithKline. JVM has received honoraria for consulting from Medtronic Spine LLC and Synthes. LB has received honoraria for consulting from Medtronic Spine LLC. DW has received honoraria for consulting from Medtronic Spine LLC and Cryolife, and has received research funding from Medtronic Spine LLC, Zimmer, Apatec, and Cryolife.

## Authors' contributions

JR made a substantial contribution to the conception and design of the study, and drafting the manuscript. AT made a substantial contribution to the analysis and interpretation of data, and drafting the manuscript. SB, JVM and LB made a substantial contribution to the analysis and interpretation of data, and critically revising the manuscript for intellectual content. DW made a substantial contribution to acquisition of data and critically reviewed the manuscript for intellectual content. All authors have read and approved the final manuscript.

## Pre-publication history

The pre-publication history for this paper can be accessed here:

http://www.biomedcentral.com/1471-2288/12/35/prepub
